# SF_6_ Optimized O_2_ Plasma Etching of Parylene C

**DOI:** 10.3390/mi9040162

**Published:** 2018-04-02

**Authors:** Lingqian Zhang, Yaoping Liu, Zhihong Li, Wei Wang

**Affiliations:** 1 Institute of Microelectronics, Peking University, Beijing 100871, China; zlqpku@gmail.com (L.Z.); yaopingliu@gmail.com (Y.L.); zhhli@pku.edu.cn (Z.L.); 2 National Key Laboratory of Science and Technology on Micro/Nano Fabrication, Beijing 100871, China

**Keywords:** Parylene C, SF_6_, O_2_ plasma etching

## Abstract

Parylene C is a widely used polymer material in microfabrication because of its excellent properties such as chemical inertness, biocompatibility and flexibility. It has been commonly adopted as a structural material for a variety of microfluidics and bio-MEMS (micro-electro-mechanical system) applications. However, it is still difficult to achieve a controllable Parylene C pattern, especially on film thicker than a couple of micrometers. Here, we proposed an SF_6_ optimized O_2_ plasma etching (SOOE) of Parylene C, with titanium as the etching mask. Without the SF_6_, noticeable nanoforest residuals were found on the O_2_ plasma etched Parylene C film, which was supposed to arise from the micro-masking effect of the sputtered titanium metal mask. By introducing a 5-sccm SF_6_ flow, the residuals were effectively removed during the O_2_ plasma etching. This optimized etching strategy achieved a 10 μm-thick Parylene C etching with the feature size down to 2 μm. The advanced SOOE recipes will further facilitate the controllable fabrication of Parylene C microstructures for broader applications.

## 1. Introduction

**Figure 1:**
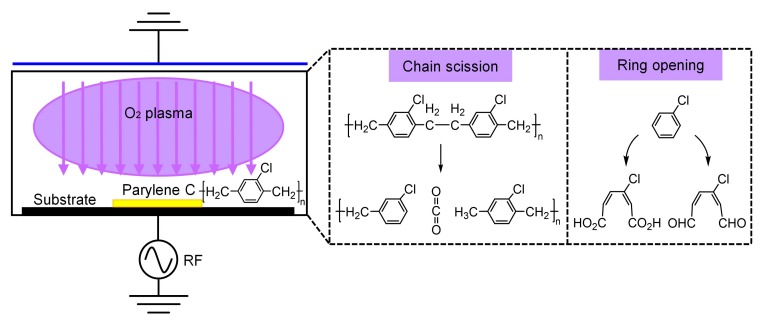
Principle of the O_2_ plasma etching of Parylene C, showing the schematic of a reactive ion etching system and representative chemical processes of Parylene C removal. During the Parylene C etching, the polymer chain scission occurred with carbon dioxide as the etch product, then aromatic ring was broken to form either aldehyde groups or carboxylic groups on the resultant chain [11,21,22].

Parylene C, or poly(monochloro-p-xylylene), is one of the most-used polymer materials in MEMS (micro-electro-mechanical system) for its compatibility with microfabrication techniques and excellent material properties. It is a chemically stable, USP (United States Pharmacopeia) Class VI biocompatible, and flexible material that has been widely implemented in microfluidics and bioMEMS applications such as microvalves [1], accelerometers [2], flexible electrodes [3,4], and neural probes [5,6,7]. To pattern the Parylene C, different fabrication techniques have been proposed, such as wet etching using chloronapthelene or benzoyl benzoate [8], dry etching based on O_2_ plasma [9,10,11,12,13,14,15], thermal imprinting or micro-molding [16,17] and laser micromachining [18,19,20]. Among the existing fabrication strategies, the dry etching technique is a relatively clean and effective method that is suitable for batch fabrication of Parylene C microstructures. Therefore, the O_2_ plasma removal of Parylene C has been investigated and widely used [9,10,11,12,13,14,15,21,22,23,24,25] (Figure 1). These studies presented the effects of process parameters such as mask material, temperature, gas flow rate and power on the etching performance. However, fabrication of controllable Parylene C patterns with small feature sizes for a thick film, i.e., with high aspect ratios, still calls for optimized etching approaches. Various methods such as switching chemistry plasma etching by deep reactive ion etching (DRIE) [26], O_2_ plasma etching with aluminum or nickel hard mask [27,28] and O_2_ plasma etching with thick negative photoresist mask [28] have been reported. Although these methods have achieved Parylene C microstructures for the specific devices, problems such as low geometrical resolution limited by the thick photoresist mask and Parylene C residuals during the metal masked etching are still not fully solved.

In this work, we attempted to address these problems by developing an SF_6_ optimized O_2_ plasma etching (SOOE) strategy using titanium as the etching mask. The titanium hard mask can achieve higher pattern accuracy and etching selectivity than the commonly used photoresist for deep Parylene C etching, and the added SF_6_ can remove the residuals caused by the sputtering effects of titanium mask during the O_2_ plasma etching. Compared with other reported etching methods, this SOOE strategy has the merits of controllability for high aspect ratio Parylene C patterning without residuals.

## 2. Materials and Methods

### 2.1. Parylene C Preparation

A 4-inch Si wafer (100, p-type) was prepared as the deposition substrate. The Parylene C used in this study was chemical vapor deposited with a commercial coating system (PDS 2010, Specialty Coating Systems Inc., Indianapolis, IN, USA). The deposition process consisted of three main steps. First, the powder-like Parylene C dimer was vaporized at approximately 175 °C under vacuum. The evaporated dimer was then pyrolyzed to radical Parylene C monomers at 690 °C. Finally, the monomer vapor entered the room temperature deposition chamber, where it polymerized onto all the exposed surfaces. The deposition pressure was 21 mTorr. 10 μm-thick Parylene C film was prepared with the loaded 16 g dimer.

### 2.2. Mask for Etching

**Figure 2:**
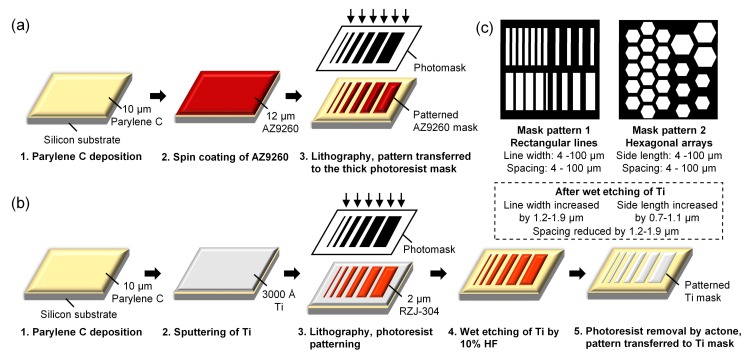
The fabrication process of the etching masks. (**a**) Fabrication of 12 μm AZ4620 photoresist mask; (**b**) Fabrication of 3000 Å titanium mask; (**c**) Schematic of the mask patterns and the corresponding dimensions. Mask pattern 1 was used for the etching by pure O_2_ plasma and mask pattern 2 was used for the SF_6_ optimized O_2_ plasma etching.

Figure 2a,b schematically showed the fabrication process of the thick photoresist mask and titanium hard mask on Parylene C. AZ9260 (AZ Electronic Materials, Branchburg, NJ, USA) was used as the thick photoresist etching mask. After priming the Parylene C substrate with hexamethyldisilazane (HMDS), 12 μm of AZ9260 was spin coated by SSE Spin Coater with 600 rpm for 4 s followed by 1500 rpm for 60 s. After baking at 98 °C for 15 min, exposure for 200 s, and development for 1 min, the patterned photoresist was prepared on the Parylene C film for the following O_2_ plasma etching. Titanium was used as the metal hard mask for its small surface stress, low cost and good adhesion with Parylene C. 3000 Å titanium was sputtered on the Parylene C film by Research s-Gun II (Sputtered Films, Santa Barbara, CA, USA). The titanium pattern was then generated by a regular photolithography using a positive photoresist (RZJ-304, Ruihong Electronic Chemical Company, Suzhou, China), following with a wet etching step by 10% HF for 40 s and a photoresist removal step by acetone.

As shown in Figure 2c, two kinds of the mask patterns were used in this work. The mask pattern 1, including rectangular lines with line width and spacing ranging from 4 to 100 μm, was applied to the pure O_2_ plasma etching. The mask pattern 2, including hexagonal arrays with side length and spacing ranging from 4 to 100 μm, was used for the SF_6_ optimized O_2_ plasma etching. For the titanium mask, due to the undercut of 0.6–0.9 μm caused by the wet etching process, the corresponding feature sizes of the patterns changed by approximately 1 μm after the fabrication, as listed in Figure 2c. The etching loading (exposed area of the wafer relative to the total wafer area) was approximately 31% for the 4-inch wafer.

### 2.3. Dry Etching Conditions

Parylene C etching was performed by a reactive ion etching (RIE) system (ME-6A, Chinese Academy of Sciences, Beiijng, China) in this study. Test samples were etched under varied process conditions including etching power ranging from 150 W to 350 W, different O_2_ gas flow rates (50–65 sccm) and SF_6_ gas flow rates (0–8 sccm). Etches were composed of repeated etching cycles, and the etching time of each cycle was set as 5 min to avoid the thermal effects and nonuniformity caused by the long-term etching. This was because sustained ion bombardment at longer etching time would result in a higher temperature on the wafer surface, which may lead to stress problems for the mask, loss of etching anisotropy and more etching nonuniformity.

### 2.4. Etching Performance Measurements

**Figure 3:**
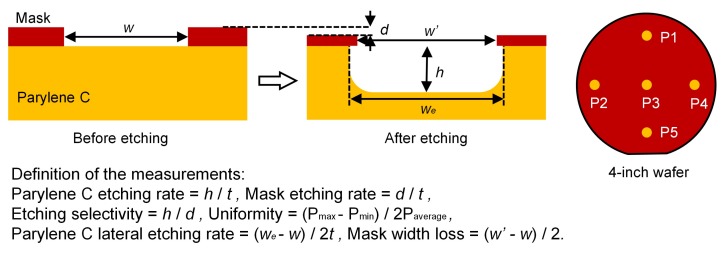
Schematic of the etching measurements.

To characterize the etching performance, etching depth and film thicknesses were measured using a profilometer (AS-500, KLA-Tencor, Milpitas, CA, USA) and a thin film-thickness measurement system (ST-2000, K-MAC, Daejeon, Korea). As shown in Figure 3, etching parameters such as Parylene C etching rate, mask etching rate, etching selectivity and uniformity were extracted from the measurements. Measurements were made over 4-inch silicon wafers at five fixed points across the sample for statistical analysis. Morphologies of the etched Parylene C were observed using a high-resolution scanning electron microscope (FEI Quanta 200 FEG, FEI Company, Hillsboro, OR, USA). To determine the elements on the etched surface and residuals, analysis on SEM-EDX experiments were performed.

## 3. Results and Discussions

### 3.1. Reactive Ion Etching of Parylene C by Pure O_2_ Plasma

Firstly, the reactive ion etching of Parylene C by pure O_2_ plasma was performed for optimizing the etching parameters of the etching system. The etching measurements including etching rate, selectivity, and uniformity were investigated under different etching parameters such as mask, gas flow and power. As summarized in Table 1, it was clear that the Parylene C etching rate increased from 218.4 nm/min to 435.4 nm/min with the increment of the plasma power (from 150 W to 350 W) and oxygen flow rate (from 50 sccm to 65 sccm). Using titanium as the etching mask, we obtained 56.4 nm/min and 74.9 nm/min slower Parylene C etching rates compared with the photoresist mask under the conditions of 350 W, 60 sccm and 250 W, 60 sccm. This may be attributed to the micro-masking effect of the residuals during the etching. Neither the etching selectivity nor the uniformity showed a clear correlation with the flow rate and etching power. The photoresist was etched off at a similar rate to Parylene C, while the titanium maintained a high etching selectivity over 100 under pure O_2_ plasma etching. All of the etching recipes achieved a good etching uniformity ranging from 1% to 3.7% (less than 5%).

**Figure 4:**
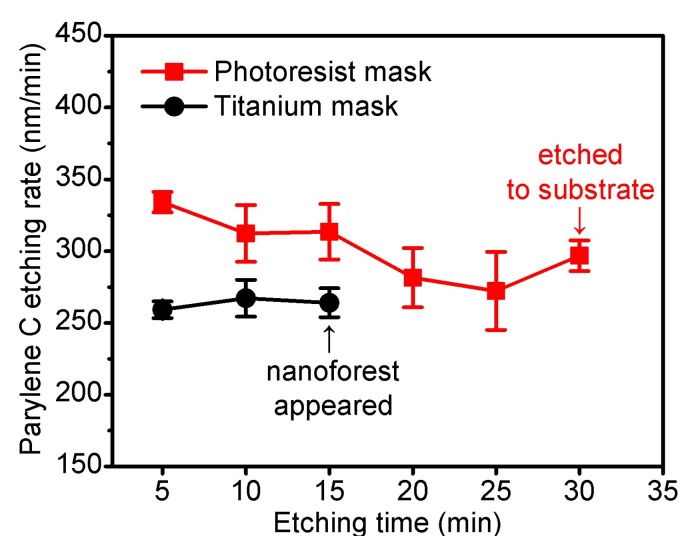
Etching rates of Parylene C during continuous pure O_2_ plasma etching using photoresist and titanium masks (n = 5, error bars represent SD). For the photoresist mask, Parylene C films were etched to the silicon substrate after 6 cycles; for the titanium mask, nanoforest structures appeared on the etched Parylene C surface after 3 cycles.

Then, continuous etching of the 10 μm-thick Parylene C films were performed. It was found that although increasing the power and gas flow rate could achieve a fast etching rate, it can also generate heavier thermal loads and even result in a crumpled mask. Temperature tests were performed by the surface temperature indicating strips (THERMAX, TMC Hallcrest, Connah’s Quay, UK) as a reference for the highest temperature during the etching. The results showed that the surface temperature was up to 149 °C under continuous etching of 350 W power, 60 sccm O_2_ flow, while for the 250 W power, 60 sccm O_2_ group, the highest surface temperature was 121 °C. Within the range of the above parameters, 250 W power, 60 sccm O_2_ flow showed the best etching performances during continuous etching of 6 cycles. The etching rates of Parylene C during continuous 250 W, 60 sccm O_2_ plasma etching were shown in Figure 4.

**Figure 5:**
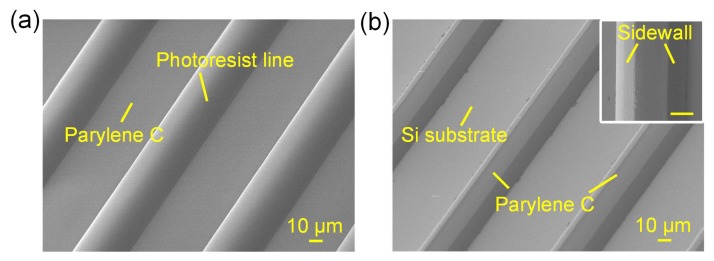
Surface morphology of the patterned photoresist and the Parylene C after O_2_ plasma etching. (**a**) Patterned 12 μm-thick AZ9260 photoresist on the Parylene C film; (**b**) Etched Parylene C structure after photoresist removal, with inset showing the sidewall, scale bar = 10 μm.

Under the 12 μm-thick AZ9260 photoresist mask, 10 μm Parylene C films were successfully etched off till the silicon substrate was exposed, as shown in Figure 5. Limited by the etching selectivity, the photoresist mask had to be thick enough, which increased the process difficulty and reduced the pattern resolution. It was not easy to achieve a steep sidewall for the thick photoresist (thickness larger than 10 μm), which would lead to an unavoidable mask width loss and the dimension variation between photoresist mask and Parylene C. The calculated total mask width loss was approximately 7 μm for the Parylene C etching.

**Figure 6:**
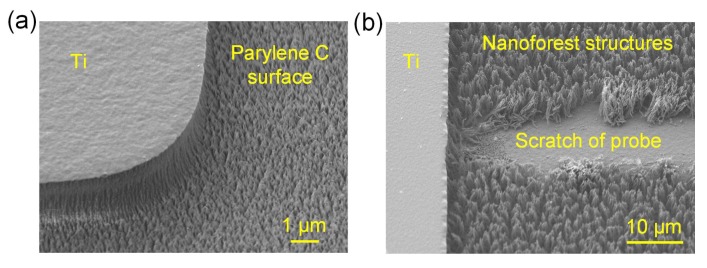
Surface morphology of Parylene C after pure O_2_ plasma etching using titanium mask. (**a**) Structure with Parylene C nanoforest after three cycles of 5 min etching; (**b**) Nanoforest scratched by the probe,:the Parylene C was etched after six cycles of 5 min pure O_2_ plasma etching.

For the titanium mask, after three cycles of etching, the nanoforest structures appeared on the etched surface of Parylene C and prevented further etching. Even after several subsequent cycles of O_2_ plasma etching, the nanoforest still existed. The surface morphology of the etched Parylene C structure is shown in Figure 6. Tests showed that the probe of the profilometer could scratch the nanoforest off the etched surface. The nanoforest structures were inferred as residuals during etching, which was also reported in Parylene C etching with aluminum or nickel metal masks [27,28], the photoresist etching with different substrates [29,30] and the polymeric etching with different reactor wall materials [31,32,33].

**Figure 7:**
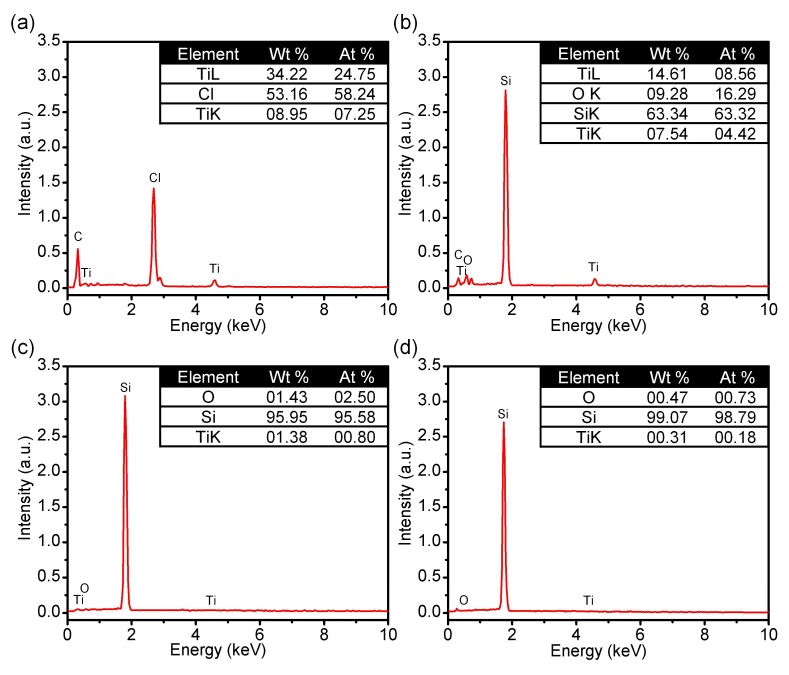
SEM-EDX results of the Parylene C etching structure on different focused area. (**a**) Patterned titanium area; (**b**) Top of the Parylene C forest; (**c**) Bottom of the Parylene C forest; (**d**) Silicon substrate after peeling the Parylene C off.

To further investigate the origins of the observed Parylene C nanoforests, we performed SEM-EDX experiments to characterize the element distribution on the patterned titanium area, top and bottom area of Parylene C etching surface, and silicon substrate after peeling the Parylene C film off. As shown in Figure 7, the element analysis showed an unexpected titanium on the etched Parylene C area, which would not appear unless the titanium mask was sputtered by the O_2_ plasma. It can be inferred that the nanoforest was attributed to the micro-masking effect caused by the titanium mask sputtering. This inference was also supported by the reported formation process of nanotexturing by plasma etching, that the sputtering of reactor wall material would create micro-masking for different kinds of nanostructures [31,32,33]. The existence of sputtered wall metal material on the etched surface was verified by the X-ray photoelectron spectroscopy (XPS) analysis in Ref. [33], showing the cause of the micro-masking formation which led to the development of nanotexture. Similarly, in this work, the titanium mask was supposed to be sputtered under the O_2_ plasma bombardment, and then deposited on the Parylene C etched surface as the micro-mask for the nanoforest formation. After the formation of nanoforest structures on the etched Parylene C area, the etching rate dropped dramatically due to its morphology. As a result, the nanostructures on the Parylene C finally prevented the plasma etching from proceeding normally.

### 3.2. SF_6_ Optimized O_2_ Plasma Etching (SOOE)

**Figure 8:**
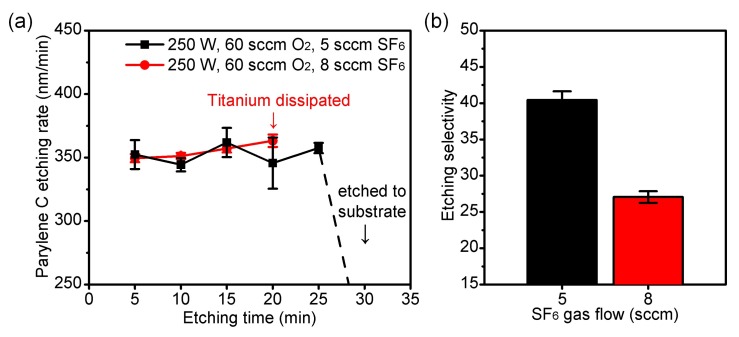
Measurements of the etching by the SOOE process with titanium as the etching mask (n = 5, error bars represent SD). (**a**) Etching rates of Parylene C during continuous SOOE; (**b**) Etching selectivity of Parylene C to mask using 250 W, 60 sccm O_2_ plasma and SF_6_.

To prevent the formation of the nanoforest structures, we added a small flow rate of fluorine-based gas, SF_6_, into the O_2_ plasma etching of Parylene C in this work. The SF_6_ could be dissociated and excited to fluorine free radicals during the process, which turned the metallic compound into metal fluoride. Therefore, during the O_2_ plasma etching, the SF_6_ could simultaneously remove the titanium or titanium oxide micro-masks on the etched surface and keep the etching proceeding. Because the fluorine free radicals also react with the metal mask, the SF_6_ gas flow should be controlled within a proper range to keep a high selectivity. We performed the measurements for SF_6_ optimized O_2_ plasma etching (SOOE) under 250 W, 60 sccm O_2_ with SF_6_ flow rate from 5 to 8 sccm, as summarized in Table 2. The etching rates of Parylene C were basically the same as approximately 350 nm/min with uniformity of less than 5% for the SOOE groups, while the etching selectivity showed a significant reduction from 40.4 to 27.1 when increasing the SF_6_ flow from 5 sccm to 8 sccm. Compared with the titanium masked etching without the SF_6_, the etching rates of the SOOE groups showed an obvious increment, implying micro-mask removal by SF_6_. Continuous etching was also performed under 250 W, 60 sccm O_2_ with SF_6_ flow rate from 5 to 8 sccm, as shown in Figure 8. The reduced selectivity under 8 sccm SF_6_ resulted in the dissipation of metal mask after continuous etching.

**Figure 9:**
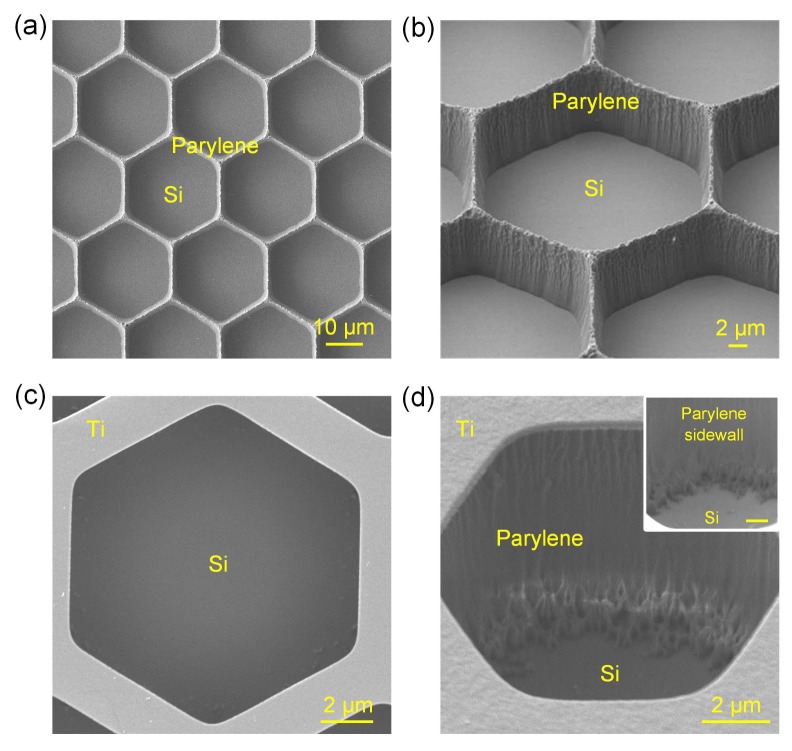
SEM of the etched Parylene C structures with the SOOE process. (**a**,**b**) Structures with 2 μm hexagon line patterns; (**a**) top view; (**b**) oblique view after tilting the structures with 45°; (**c**,**d**) Structures with 5 μm hexagon pore patterns; (**c**) top view; (**d**) oblique view after tilting the structures with 45°, with inset showing the zoomed bottom area, scale bar = 1 μm.

With SOOE etching parameters of 250 W, 60 sccm O_2_ and 5 sccm SF_6_, 10 μm Parylene C films were successfully etched off till the silicon substrate was exposed. Both the Parylene C hexagon line patterns with width of 2 μm (corresponding photomask line width of 4 μm) and hexagon pore patterns with side length of 5 μm (corresponding photomask side length of 4 μm) were controllably etched with no obvious residuals, as shown in Figure 9. The etching also showed a relatively steep sidewall of 85° with Parylene C lateral etching rate of 27 nm/min.

The comparison of Parylene C etching performance using different metal masks is briefly shown by Table 3. It is clear from the table that, without the SF_6_, the etching using aluminum, nickel or titanium masks faced the same situation: noticeable residuals were created in the openings. By introducing a 5-sccm SF_6_ flow in this work, the residuals were effectively removed during the O_2_ plasma etching, and the 10 μm-thick Parylene C etching with feature size down to 2 μm was achieved. It was also worth mentioning that the titanium, which was commonly used as a metal adhesion layer on the Parylene C in device fabrication, could achieve better adhesion than the aluminum or nickel masks and could be easily wet etched by 10% HF in room temperature, which would facilitate the mask patterning with smaller feature sizes on the Parylene C.

## 4. Conclusions

In summary, this work developed an SF_6_ optimized O_2_ plasma etching (SOOE) of Parylene C. This method overcame the challenges existing in the pure O_2_ plasma etching of thick Parylene C film, i.e., low geometrical resolution when using photoresist as the etching mask and the nanoforest residuals when using metal as the etching mask. The SF_6_ effectively removed the nanoforest residuals caused by sputtered metal micromask, without an increase of the fabrication complexity. The results showed an excellent 10 μm Paryene C etching under the recipe of 250 W, 60 sccm O_2_ and 5 sccm SF_6_, with line width down to 2 μm. The developed SOOE process will further facilitate the controllable fabrication of Parylene C microstructures for a variety of applications in microfluidic, bio-sensing or implantable devices.

## Figures and Tables

**Table 1:** Summary of the etching recipes and measurements for reactive ion etching of Parylene C by pure O_2_ plasma.

Parameters			Measurements			
Mask Type	O_2_ Flow (sccm)	Power (W)	Parylene C Etching Rate (nm/min)	Mask Etching Rate (nm/min)	Selectivity	Uniformity
Photoresist	60	150	218.4 ± 5.9 ^1^	199.4 ± 17.1	1.10 ± 0.09	3.7%
	60	250	334.2 ± 7.1	321.1 ± 34.9	1.05 ± 0.11	2.6%
	60	350	414.7 ± 4.7	426.9 ± 24.8	0.97 ± 0.05	1.4%
	50	350	359.9 ± 5.5	366.7 ± 61.8	1.00 ± 0.17	2.1%
	55	350	381.0 ± 12.5	392.1 ± 14.9	0.97 ± 0.05	1.7%
	65	350	435.4 ± 4.2	445.2 ± 14.2	0.98 ± 0.04	1.0%
Titanium	60	350	358.3 ± 3.6	<20	>100	1.0%
	60	250	259.3 ± 5.9	<20	>100	2.8%

^1^ Data represent mean ± standard deviation (S.D.), n = 5, the same as below.

**Table 2:** Summary of the etching recipes and measurements for SF_6_ optimized O_2_ plasma etching (SOOE).

Parameters				Measurements			
Mask Type	O_2_ Flow (sccm)	SF_6_ Flow (sccm)	Power (W)	Parylene C Etching Rate (nm/min)	Mask Etching Rate (nm/min)	Selectivity	Uniformity
Titanium	60	0	250	259.3 ± 5.9 ^1^	<2	>100	2.8%
	60	5	250	352.4 ± 11.4	8.7 ± 0.5	40.4 ± 1.1	3.9%
	60	8	250	349.6 ± 3.2	13.0 ± 0.7	27.1 ± 0.8	1.1%

^1^ Data represent mean ± standard deviation (S.D.), n = 5, the same as below.

**Table 3:** The comparison of Parylene C etching performance using different metal masks.

Mask Type	Etching Method	Minimum Feature Size	Parylene C Thickness	Aspect Ratio	Residuals
Aluminum (Ref. [27])	O_2_ plasma, ICP (inductively coupled plasma)	6 μm	10–55 μm	9:1	Unavoidable residuals on the substrate (>1 μm in width & height)
Nickel (Ref. [28])	O_2_ plasma, ICP-RIE	50 μm	23 μm	1:2	Residuals found in the opening & prevented the carved Parylene pieces from proper peeling
Titanium (this work)	O_2_ plasma, RIE	2 μm	4 μm (not etched to substrate)	2:1	Nanoforest residuals appeared on the surface & prevented the etching from proceeding normally
	SF_6_ added (SOOE)	2 μm	10 μm	5:1	Residual free etching was achieved
